# Nutcracker Phenomenon: A Rare Incidental Finding

**DOI:** 10.7759/cureus.32822

**Published:** 2022-12-22

**Authors:** Ghaida B AlQefari, Khalil I Alduraibi, Abdulwahab A Almansour, Asail Alghamdi, Mohammed A Alsubhi

**Affiliations:** 1 General Medicine, Qassim University College of Medicine and Surgery, Qassim, SAU; 2 General Medicine, King Saud University College of Medicine and Surgery, Riyadh, SAU; 3 General Medicine, King Saud University College of Medicine, Riyadh, SAU; 4 College of Medicine, Albaha University, Albaha, SAU; 5 Radiology, Albaha University Faculty of Medicine, Albaha, SAU

**Keywords:** incidental, rapid weight loss, nutcracker syndrome, left renal vein entrapment syndrome, nutcracker phenomenon

## Abstract

The nutcracker phenomenon, or left renal vein (LRV) entrapment syndrome, occurs when there is compression of the LRV, mostly between the abdominal aorta and the superior mesenteric artery. Patients with nutcracker syndrome (NCS) may present with various symptoms, with the most common being hematuria, left flank pain, varicocele in males, proteinuria, and anemia. Our 22-year-old male patient presented with abdominal pain without hematuria. Insidiously, we made the diagnosis of NCS with this unusual presentation. Some studies have proposed a relationship between rapid weight loss in a short period of time and the appearance of NCS. We recommend that healthcare providers suspect NCS in patients who present with abrupt severe abdominal discomfort, particularly in situations that coincide with rapid weight loss for an unknown reason.

## Introduction

The nutcracker phenomenon, or left renal vein (LRV) entrapment syndrome, occurs when there is compression of the LRV mostly between the abdominal aorta (AA) and the superior mesenteric artery (SMA). Nutcracker syndrome (NCS) is often described in patients who present with clinical symptoms related to nutcracker anatomy [1]. The nutcracker phenomenon refers to the anatomic findings suggestive of NCS in asymptomatic patients [2]. NCS is believed to be a rare disorder despite the absence of precise prevalence and incidence rates, with reported occurrences varying by age group [3]. Previously reported studies have described patients ranging from infancy to those in the seventh decade of life [4,5]. The prevalence peaks in middle-aged adults during the second and third decades, most likely because of the rapid development of the vertebral bodies during adolescence, which causes the angle between the AA and SMA to shrink [5]. According to previous studies, males are diagnosed (23.59 ± 13.09 days) earlier than females (29.34 ± 13.93 days) [6]. NCS is categorized into two types, namely, anterior and posterior. In anterior NCS, the normally located LRV is compressed by the AA and SMA. In posterior NCS, the retro-aortic LRV is compressed usually between the AA and vertebral column [2]. Patients with NCS may present with various symptoms, with the most common symptoms including hematuria, left flank pain, varicocele in males, proteinuria, and anemia [6]. Patients may also present with abdominal pain, dysmenorrhea, dyspareunia, orthostatic hypotension, fatigue, infertility, and varicose veins of the abdomen, vaginal wall, buttocks, or upper thighs [2,7,8]. In rare cases, autonomic dysfunction symptoms, such as hypotension, tachycardia, and syncope, have been reported [9].

NCS is initially diagnosed by excluding other causes and diseases that may precipitate to the patient’s current complaint. Complete information including history, physical examination findings, and lab investigations, such as urine analysis, urine culture, and urine phase-contrast microscopy, should be collected. Further, the presence of macroscopic and microscopic hematuria and proteinuria should be evaluated. Imaging of the kidneys should be done including Doppler ultrasonography (DUS), computed tomography angiography (CTA), magnetic resonance imaging (MRI), retrograde venography, and intravascular ultrasound (IVUS) [2,5]. The gold standard for the diagnosis of NCS is venography with measurement of the renocaval pressure gradient, which is usually unnecessary for the diagnosis due to its invasiveness [4]. Recent studies have recommended CT due to its accuracy and ability to investigate abdominal findings. Of the CT parameters, the most accurate finding is the beak sign and LRV diameter ratio (hilar-aortomesenteric) ≥4.9 [5,10]. There is controversy regarding the management of NCS, both in terms of good indications for the treatment, as different diagnostic methods are utilized, and the best treatment modality for each patient. In asymptomatic cases or cases with mild symptoms or mild hematuria, conservative management is preferred [11,12]. However, in cases with gross hematuria (especially if recurrent), severe symptoms, such as flank pain and abdominal pain, anemia, autonomic dysfunction, impairment of renal function, such as persistent orthostatic proteinuria, and varicocele formation, surgery may be indicated. Surgery may also be indicated in patients in whom conservative management has failed after 24 months in those less than 18 years of age and after six months in adults [12-22].

## Case presentation

A 22-year-old male presented to the emergency department complaining of abdominal pain for three hours. The patient was at home when the pain started. The pain did not start during an activity or eating. It was located in the umbilical and suprapubic abdominal areas, was colicky in nature, and did not radiate elsewhere. Moreover, the pain was continuous, started suddenly, and was initially progressive but then continued in the same severity. The patient tried paracetamol and eating or drinking with no improvement in pain. He reported that the pain aggravated with food and water intake, as well as on changing positions or remaining in one position. The patient had never experienced similar pain before. He rated the pain as 10/10. He tried to sleep but could not because of the pain. He also reported an inability to move because of the pain.

There was no history of eating out, fever, diarrhea, blood or mucus with stool, nausea, bloating, jaundice, pruritus, regurgitation or heartburn, dyspepsia or painful defecation, and urinary and respiratory symptoms. In addition, there was no history of contact with sick patients or recent travel. The patient was following a strict diet and an exercise plan and reported an intentional fat loss of 11 kg, as measured by a digital scale (lost 6 kg in body weight), in the last three to four months. The patient was a non-smoker. His family history was unremarkable. On arrival, his vitals were recorded (Table 1) and a physical examination was done. Laboratory routine chemistry and general initial investigations are shown in Table 2 and Table 3.

**Table 1:** Vital signs on arrival.

Variable	Result	Normal range
Blood pressure (mmHg)	133/86	90/60–120/80
Heart rate (beats per minute)	56	60–100
Respiratory rate (breaths per minute)	19	12–16
Temperature (°C)	37.0	36.1–37.2
Oxygen saturation	99% on room air	95–100%

**Table 2:** Routine chemistry findings.

Variable	Result	Normal range
Blood urea nitrogen (mmol/L)	7.5 (H)	2.5–6.4
Creatinine (µmol/L)	84	62–115
CO_2_ (mmol/L)	28	21–32
Chloride (mmol/L)	101	95–110
Glucose (mmol/L)	7.2 (H)	3.9–5.8
Potassium (mmol/L)	3.5	3.5–5.1
Sodium (mmol/L)	136	135–145
Osmolality (mOsm/kg)	287	285–295

**Table 3:** General hematology findings. WBC = white blood cell; RBC = red blood cell; MCV = mean corpuscular volume; MCH = mean corpuscular hemoglobin; MCHC = mean corpuscular hemoglobin concentration; RDW = red cell distribution width; MPV = mean platelet volume; NRBC = nucleated red blood cell

Variable	Result	Normal range
WBC (×10^9^/L)	4.1	4.0–11.0
RBC (×10^12^/L)	6.1	4.7–6.1
Hemoglobin (g/L)	165	130.0–180.0
Hematocrit (%)	49.2	42.0–52.0
MCV (fL)	80.3	80.0–94.0
MCH (pg)	26.9 (L)	27.0–32.0
MCHC (g/L)	335.0	320.0–360.0
RDW (%)	13.9	11.5–14.5
Platelet (×10^9^/L)	228	140.0–450.0
MPV (fL)	6.7 (L)	7.2–11.1
Neutrophil auto (×10^9^/L)	2.3	2.0–7.5
Neutrophil auto (%)	56.5	40.0–75.0
Lymphocyte auto (×10^9^/L)	1.6	1.0–5.0
Lymphocyte auto (%)	37.6	20.0–45.0
Monocyte auto (×10^9^/L)	0.2	0.2–0.8
Monocyte auto (%)	3.8	3.0–9.0
Eosinophil auto (×10^9^/L)	0.00	0.00–0.80
Eosinophil auto (%)	1.0	0.0–6.0
Basophil auto (×10^9^/L)	0.00	0.00–0.20
Basophil auto (%)	1.10 (H)	0.00–1.00
NRBC	0.0000	0.0000–0.0001

Physical examination

On physical examination, the patient was conscious, alert, and oriented, He was in pain and could not walk comfortably. Cardiovascular examination showed normal heart sound, regular rate and rhythm, and no murmurs or added sounds. The chest was clear to auscultation bilaterally, with equal air entry and no added sounds. On abdomen palpation, tenderness was noted in the umbilical and suprapubic areas. His abdomen was soft and lax without rebound, guarding, or rigidity. Per rectum examination showed no masses or tenderness. There were no neurological focal deficits. His skin was warm and dry without any rashes.

Differential diagnoses

The differential diagnoses for acute pain in the periumbilical and suprapubic areas included pancreatitis, peptic ulcer disease, mesenteric ischemia, small bowel obstruction, umbilical hernia, gastroenteritis, diverticulitis, early appendicitis presenting with periumbilical pain, cystitis, and urinary tract infections.

Confirmatory investigation

**Figure 1:**
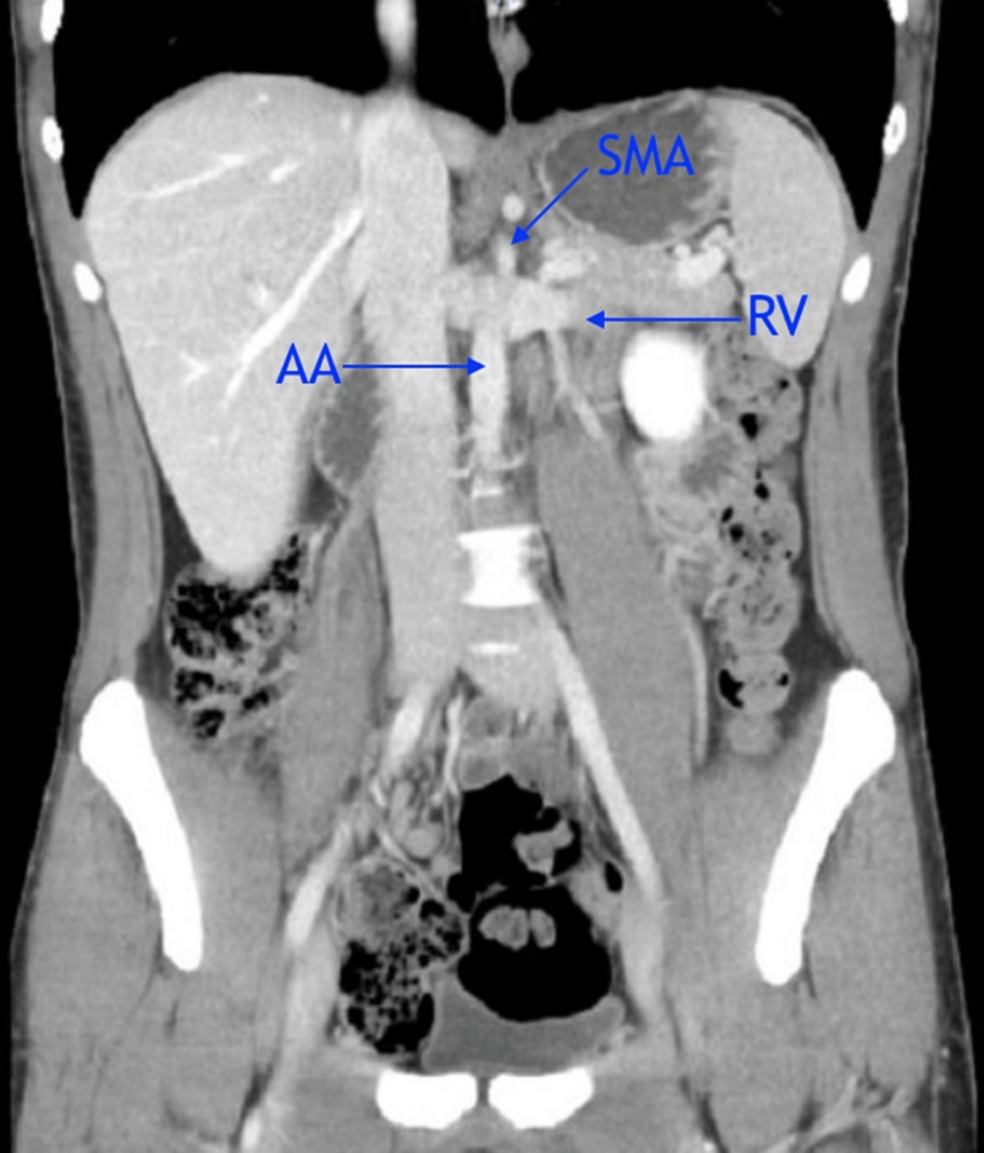
Coronal view showing the entrapment of the left RV between the AA and SMA. RV = renal vein; AA = abdominal aorta; SMA = superior mesentric artery

**Figure 2:**
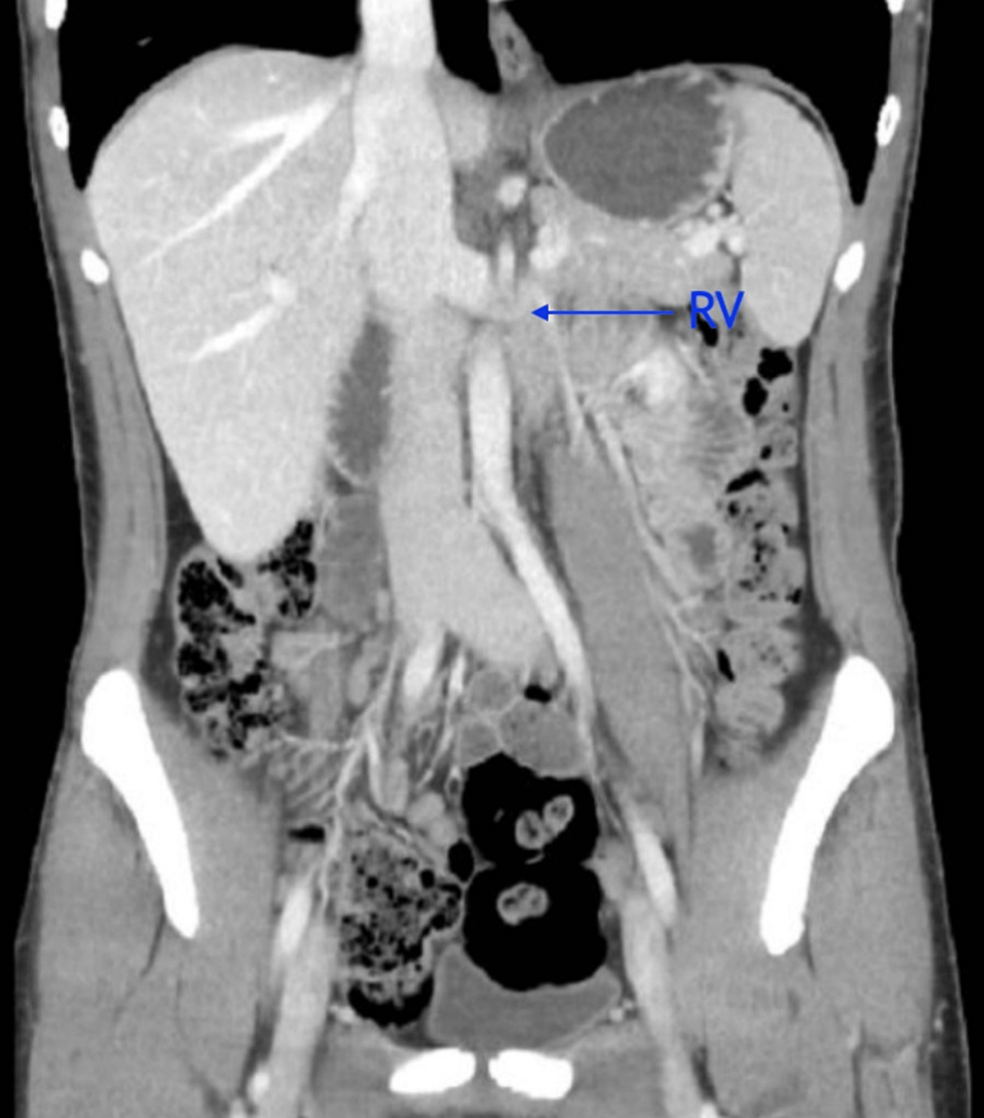
Different level of the coronal view showing the entrapment of the left RV. RV = renal vein

**Figure 3:**
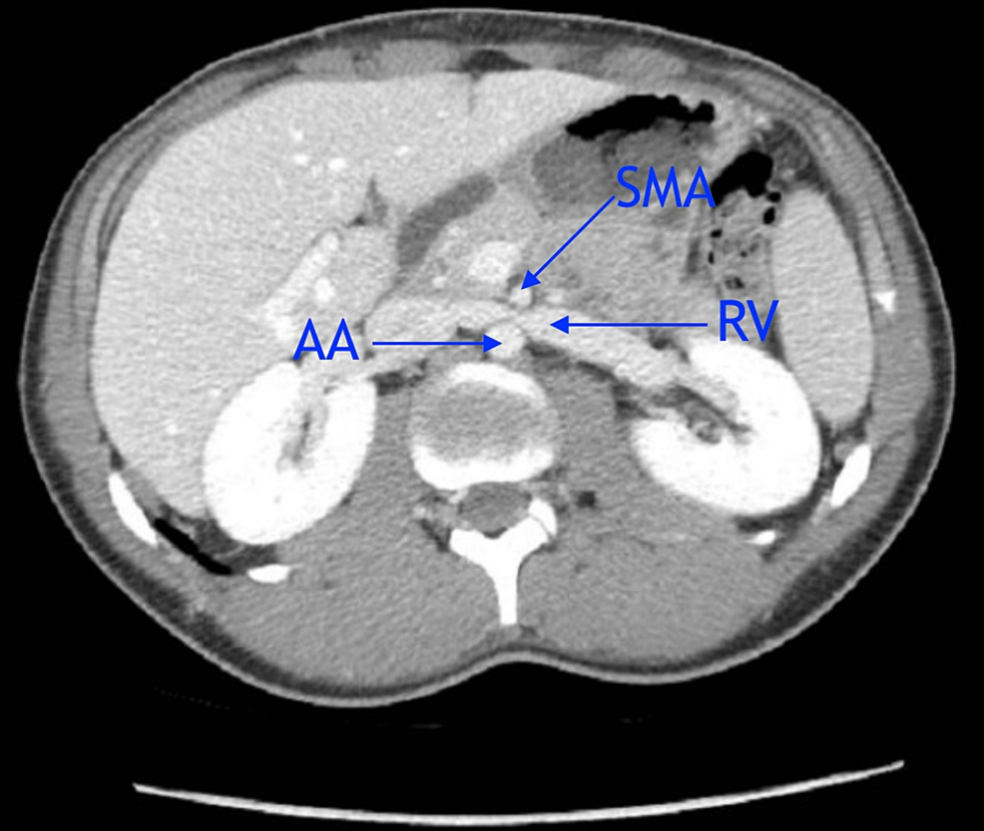
Axial view showing the left RV entrapped between the SMA and AA. RV = renal vein; SMA = superior mesentric artery; AA = abdominal aorta

A bedside US and CT of the abdomen and pelvis were conducted. The bedside US showed no signs of obstruction. On CT, the liver was homogeneously enhancing without a focal lesion. The portal vein and hepatic vessels were patent. The spleen, pancreas, adrenals, and kidneys were unremarkable. The bowel was normal in caliber. There was no lymphadenopathy in the abdomen or pelvis. There was no collection or free fluid, and no pneumoperitoneum was seen. The lung bases were clear without pleural effusion or pneumothorax. The visualized bone skeleton showed no sclerotic or lytic suspicious lesions. CT scan showed an appearance of entrapped LRV between the SMA and AA, suggestive of NCS. Figures 1-3 show the different views of the abdominal CT illustrating the entrapment of the LRV between the SMA and AA, with the otherwise normal appearance of abdominal organs, bones, and lung bases.

Diagnosis

No clear cause for the pain was identified by history, physical examination, or investigation. Hence, the final diagnosis was an incidental finding of NCS, a rare condition.

Management

The patient was managed initially by intravenous (IV) fluids, followed by analgesics. However, because the pain was not relieved by simple analgesics, the patient was administered IV morphine, following which his pain reduced substantially. Regarding the CT findings of NCS, the urology team was consulted. The patient had no symptoms of hematuria, flank pain, or orthostatic hypotension, which is an atypical presentation. He was informed and reassured about the condition.

## Discussion

Our patient mentioned that he had lost weight intentionally, and, more specifically, that he had lost 11 kg of fat, as measured by an Inbody electrical scale (6 kg weight loss by normal scale), in a period of three to four months. This is consistent with the findings of multiple studies that investigated the relationship between weight loss and the appearance of NCS symptoms.

Based on the findings of a 2017 study, one of the factors that can lead to the development of NCS is a reduction in the amount of fat in the retroperitoneal region. This can result in decreasing the angle between the AA and the SMA, which can be seen in people who lose a significant amount of weight very quickly [23]. The patient’s condition improved when he gained weight, which is consistent with the findings of another study, which indicated that weight gain can improve the symptoms of NCS [24].

Although there was no alleviating factor for the discomfort, there was an increase in pain when the position was changed. This may be because altering the position causes a change in the angle between the AA and the SMA. In most of the research conducted on NCS, hematuria has been described as one of the most typical symptoms; nevertheless, our patient did not complain of gross hematuria. In our patient, on urine analysis, his urine was clear and negative for microscopic hematuria.

We suggest performing additional studies on the relationship between weight loss and the appearance of NCS symptoms, as well as the significance of weight gain in alleviating the symptoms of NCS [7].

## Conclusions

NCS is a rare condition that appears when there is entrapment and compression of the LRV mostly between the AA and SMA. Its prevalence peaks in middle-aged adults. The most common presenting symptoms include hematuria, left flank pain, and anemia. A full approach including history, physical examination, laboratory investigations, and the presence of macroscopic or microscopic hematuria, followed by CT imaging of the kidneys, must be followed to make a diagnosis. The presence of a beak sign and LRV diameter ratio (hilar-aortomesenteric) ≥4.9 on CT imaging is the most accurate finding suggestive of NCS. The treatment of NCS depends on the severity. In asymptomatic patients or those with mild hematuria, NCS is treated conservatively. However, in severe cases with gross hematuria or in patients with autonomic dysfunction and impaired renal function, surgery is indicated. We recommend that healthcare providers raise the suspicion of NCS in patients who present with abrupt severe abdominal discomfort, particularly when the symptoms coincide with rapid weight loss if the reason is unknown.
